# Improving CTV_boost_ delineation after preoperative systemic therapy in breast cancer using deformable PET/CT registration

**DOI:** 10.1016/j.breast.2026.104752

**Published:** 2026-03-23

**Authors:** Jordy Kemmeugne, Lyse Gallay, David Morland, Judicael Hotton, Christelle Jouannaud, Yacine Merrouche, Dimitri Papathanassiou, Fabien Reyal, Marie Ndéo Sene, Paolo Torielli, Stéphane Vignot, Philippe Guilbert, Arnaud Beddok

**Affiliations:** a Department of Radiation Oncology, Institut Godinot, Reims, France; b Institut d’Intelligence Artificielle en Santé, CHU de Reims, Université de Reims Champagne- Ardenne, Reims, France; c Department of Nuclear Medicine, Institut Godinot, Reims, France; d Université Reims Champagne Ardenne, CRESTIC, Reims, France; e Department of Surgery, Institut Godinot, Reims, France; f Department of Medical Oncology, Institut Godinot, Reims, France

**Keywords:** Breast cancer, Tumor bed delineation, PET/CT, Deformable image registration, Inter-observer variability

## Abstract

**Background:**

Delineating the CTV_boost_ in postoperative breast radiation therapy (RT) may be challenging after preoperative systemic therapy (PST), due to treatment-induced anatomical changes and the potential discordance between the postoperative surgical cavity, as identified by surgical clips, and the spatial extent of the pretreatment tumor. The objective of this study was to assess whether [^18^F]-FDG PET/CT (PET/CT)-guided delineation using deformable image registration (DIR) improves inter-observer reproducibility compared to conventional strategies relying primarily on surgical clips and anatomical landmarks.

**Materials and methods:**

Fifty-eight patients treated with PST, surgery, and postoperative RT were retrospectively included. Three radiation oncologists performed four delineation strategies per patient: (1) CTV_CLI_, based on clips and anatomical landmarks; (2) CTV_PET_, a 10 mm isotropic expansion around the PET-defined biological tumor volume (BTV); (3) CTV_COM_, generated by a 15–20 mm isotropic expansion around the BTV centroid and adapted to pathological features; and (4) CTV_INT_, manually refined volumes integrating clips, PET signal, and registration accuracy. Inter-observer variability (IOV) was assessed using the Dice similarity coefficient (DSC) and Hausdorff distance (HD).

**Results:**

All patients underwent lumpectomy after PST, with a mean age of 53.9 years, and 44.2% had triple-negative tumors. The mean volume of the CTV_CLI_ was 34.4 cm^3^, compared to 36.5 cm^3^ for CTV_PET_, 21.2 cm^3^ for CTV_COM_, and 43.6 cm^3^ for CTV_INT_. Mean DSCs were 0.86 for both CTV_PET_ and CTV_COM_, 0.77 for CTV_INT_, and 0.62 for CTV_CLI_ (*p* < 10^−8^).

**Conclusion:**

PET/CT-guided delineation after DIR significantly improves inter-observer reproducibility of CTV_boost_ definition after PST compared with conventional methods.

## Highlights

• Conventional CTV_boost_ delineation shows the lowest inter-observer agreement.
• PET/CT-guided delineation improves inter-observer reproducibility of CTV_boost_.
• Integrating PET signal and anatomical context optimizes CTV_boost_ definition.

## 1 Introduction

Postoperative radiation therapy (RT) is a cornerstone of breast-conserving treatment in early and locally advanced breast cancer. In patients under 50 years of age, current French guidelines generally recommend an additional boost dose to the tumor bed (CTV_boost_), typically 10–16 Gy in standard fractionation [1,2]. Between 50 and 60 years, the boost may be omitted in selected patients with favorable prognostic features, and it is generally not recommended beyond 60 years [1]. Accurate delineation of the CTVboost, therefore, remains a key issue, particularly in younger patients who derive the greatest benefit from optimized local control.

In routine practice, however, delineating the CTV_boost_ remains a major challenge. The conventional approach relies on surgical clips marking the lumpectomy cavity on the planning CT, together with preoperative imaging and clinicopathological information [3]. Despite this multimodal basis, substantial inter-observer variability (IOV) persists, largely dependent on cavity visibility and postoperative anatomical landmarks [[4], [5], [6]]. In situations where postoperative anatomy provides only indirect surrogates of the original tumour location, this variability may be further accentuated, a limitation that is well recognized in the surgical literature and underlies the use of tumour markers placed before PST [7].

These uncertainties primarily have methodological and technical implications. A substantial proportion of ipsilateral breast tumor recurrence has been reported to occur outside the delineated tumor bed in some series [8]. Although such events may correspond to either true recurrences or new primary tumors, these observations highlight limitations in the consistency and spatial accuracy of current tumor bed delineation strategies. In the absence of robust and easily applicable guidelines, clinicians may tend to delineate larger boost volumes to reduce uncertainty, which in turn is associated with an increased risk of radiation-induced fibrosis, the principal late toxicity in this setting, with potential impact on cosmetic outcomes and quality of life [9].

In this context, the integration of metabolic imaging may represent a valuable complementary spatial reference. In patients with locally advanced breast cancer treated with PST, baseline [^18^F]-FDG PET/CT is frequently performed as part of the initial staging work-up, before any locoregional treatment [10]. Although PET/CT has limitations for assessing treatment response after PST, it provides a pre-treatment metabolic representation of the primary tumor that may remain informative for subsequent target volume definition. When fused with the planning CT using deformable image registration (DIR), PET/CT has demonstrated acceptable geometric agreement between volumes, particularly when the PET-defined volumes and the planning CT cover comparable anatomical regions [11,12]. However, no study has yet investigated whether this approach can improve inter-observer reproducibility in the delineation of the CTV_boost_.

This study aimed to evaluate whether PET/CT-based delineation using DIR has superior reproducibility compared to the standard approach.

## 2 Material and methods

### 2.1 Patient population

Adult female patients (≥18 years at diagnosis) with locally advanced breast cancer treated at XXX between June 2019 and December 2023 were retrospectively identified. Clinical and pathological staging were defined according to the AJCC 8th edition. Inclusion criteria comprised the administration of PST, the availability of a pre-treatment PET/CT, performance of breast-conserving surgery (lumpectomy), and subsequent postoperative whole-breast RT. In all cases, tumor site marking with a surgical clip was planned before PST, in accordance with national recommendations. Patients undergoing total mastectomy, lacking surgical clip placement during lumpectomy, presenting with distant tumor processes on PET/CT, or presenting non-hypermetabolic tumors were excluded. The study protocol was approved by the institutional ethics committee, and data collection was conducted following national regulations. Informed non-opposition was obtained from all patients before inclusion, in compliance with French legislation on retrospective research involving anonymized health data. Detailed information regarding PET/CT acquisition and reconstruction parameters is provided in the Supplementary Material.

### 2.2 Image registration and CTV_boost_ delineation

**Fig. 1:**
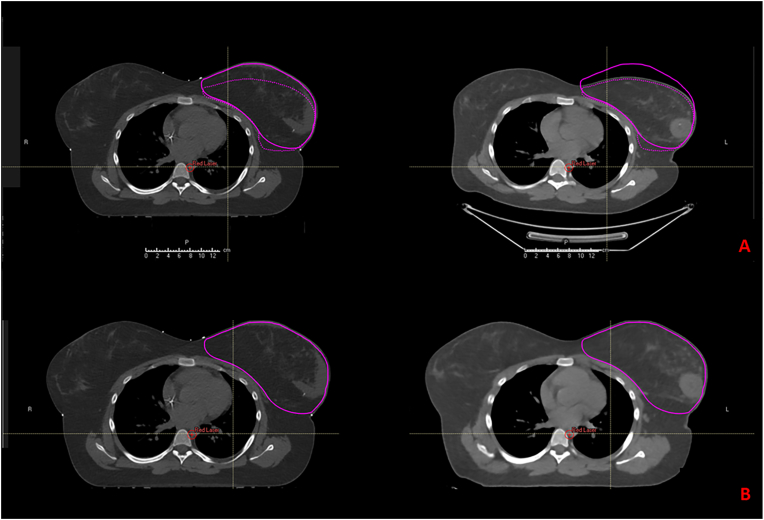
**Illustration of image fusion between PET/CT and planning CT for CTV_boost_ delineation.** A. Example of rigid image registration between the planning CT (left, “Primary”) and the CT component of the PET/CT scan (right, “Secondary”), displayed in the axial plane. Breast contours and clinical target volumes (CTV_breast_) are delineated on both image sets. The magenta solid line represents the CTV drawn on the PET/CT, and the dotted line corresponds to its projection onto the planning CT after rigid alignment. Registration was performed by visually matching thoracic structures and lateral breast borders. B**.** Example of deformable image registration between the PET/CT and the planning CT. The deformation matrix was generated based on the external contour and the CTV_breast_. This allows the PET/CT to conform more precisely to the anatomy of the planning CT. The resulting superimposition illustrates anatomical alignment between PET/CT and planning CT following deformable registration.

The DICOM series of both the planning CT and the pre-treatment PET/CT were imported into the RayStation® treatment planning system (TPS) under pseudonymized identifiers. Image registration relied on automatic segmentation of the treated breast (CTV_breast)_ on both datasets, followed by manual refinement to ensure anatomical consistency. The planning CT was used as the reference image. Rigid registration was initially performed based on thoracic wall alignment and applied to the PET images, followed by DIR using the CTV_breast_ as the region of interest to enhance local alignment (Fig. 1). Metabolic tumor volumes were visualized using a standardized 40% SUVmax threshold, in accordance with published recommendations for PET-based target delineation in breast tumors [13].

**Fig. 2:**
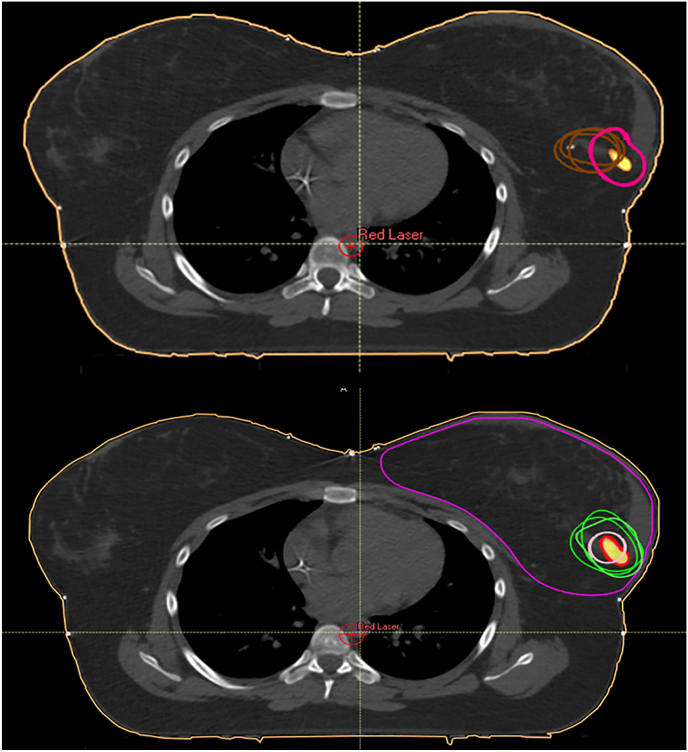
**CTV_boost_ delineation strategies following deformable PET/CT–;CT fusion**. Axial views after deformable registration using the CTV_breast_ as the registration volume. The PET-derived BTV (40% SUVmax) was transferred onto the planning CT for target construction. Top: CTV_CLI_ (brown) and CTV_PET_ (magenta). Bottom: CTV_COM_ (pink) and CTV_INT_ (green). (For interpretation of the references to colour in this figure legend, the reader is referred to the Web version of this article.)

**Fig. 3:**
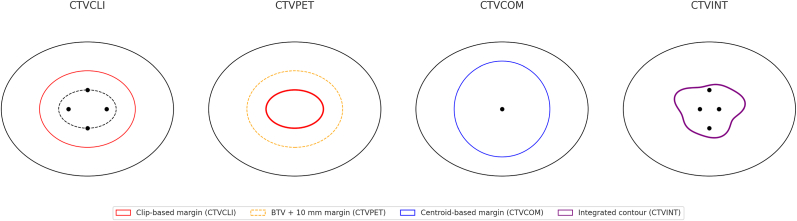
**Schematic representation of the four CTV_boost_ delineation strategies.** CTV_CLI_ (clip- and anatomy-based), CTV_PET_ (10 mm expansion of PET-defined BTV), CTV_COM_ (centroid-based expansion with ESTRO-adapted margins), and CTV_INT_ (integrative manual refinement). This schematic illustration is intended to highlight conceptual differences between approaches.

CTV_boost_ delineation was independently performed by two senior radiation oncologists,including one expert in breast RT who served as the reference, and one junior radiation oncologist. All observers were blinded to each other's contours. Four distinct contouring strategies were employed for each case. The first approach, referred to as CTV_CLI_, combined the location of surgical clips on the planning CT with preoperative imaging and surgical information, following the principles described in the GEC-ESTRO guidelines [3]. Observers defined the presumed CTV_boost_ by integrating clip positions with auxiliary data (e.g., mammography, operative notes) and by taking into account post-surgical anatomical changes. The second approach involved delineation of a biological tumor volume (BTV) on the deformably registered PET/CT. A 10 mm isotropic margin was applied to this volume to define the CTV_PET_, as a methodological construct intended to explore the spatial impact of incorporating the full pretreatment metabolic extent into boost definition, rather than as a clinically intended target volume. This margin was chosen to encompass potential microscopic spread while allowing a standardized and reproducible expansion for comparative purposes, particularly given the frequent spatial mismatch between the PET signal and the surgical cavity. The third strategy consisted of generating an isotropic expansion centered on the BTV centroid (CTV_COM_), defined as the three-dimensional geometric center of the PET-derived metabolic volume. By focusing on the tumor centroid rather than its full metabolic extent, this approach aimed to retain pretreatment spatial information while limiting unnecessary volume expansion. CTV_COM_ was therefore designed to provide a clinically more realistic surrogate of the original tumor location in situations where PST substantially modifies local anatomy, while remaining consistent with ESTRO margin principles. Margin sizes were determined according to ESTRO guidelines and tailored to the final pathological findings: 15 mm for pT0 tumors, 17 mm for pT1 R0 tumors, and 20 mm for cases with close margins (<2 mm), R1 resections, or pT2 tumors [6]. Finally, a fourth contour (CTV_INT_) was defined freely by each observer after reviewing the previously delineated volumes. This allowed critical integration of all available metabolic and anatomical information, with particular attention to image registration quality and residual tumor bed visibility (Fig. 2). All final clinical target volumes were required to be entirely enclosed within the CTV_breast_ volume. Any portion extending beyond this anatomical boundary was trimmed using Boolean tools available within the TPS. A schematic overview of the four delineation strategies is provided in Fig. 3.

### 2.3 Contour comparison and reproducibility metrics

For all patients, the delineations performed by the most experienced radiation oncologist (PG, with over 15 years of expertise in breast RT) were considered the reference standard. IOV in CTV_boost_ delineation was assessed by comparing each observer's contours to this reference, using both absolute volumetric and relative geometric indices. Absolute indices allowed for the evaluation of discrepancies in volume definition. The volume ratio (VR), defined as the ratio between the observer's contour and the reference contour, is optimal when equal to 1. The Common Delineated Volume (CDV), which quantifies the proportion of intersecting volume relative to the volume of reference, reaches optimal agreement when equal to 100%. Conversely, the Added Delineated Volume (ADV), representing the excess volume outside the intersection zone, is ideally 0%. Relative geometric indices were used to assess the spatial concordance of contours independently of their size. The Dice Similarity Coefficient (DSC) was employed to measure global volumetric overlap, with values ranging from 0 (no overlap) to 1 (perfect agreement); in clinical practice, values above 0.7 are generally considered acceptable, with a theoretical optimum of 1. The Hausdorff Distance (HD) was computed to capture the maximum surface-to-surface distance between each observer's contour and the reference. This metric is particularly sensitive to local deviations at contour boundaries and is considered acceptable below 10 mm, with the optimal value being 0 mm.

### 2.4 Statistical analysis

Descriptive statistics were used to characterize the patient population and contouring outputs. Continuous variables were summarized using mean ± SD or median (95% CI), and categorical variables as counts and percentages. For each contouring strategy, observer-to-observer differences were assessed using pairwise comparisons of predefined reproducibility metrics (VR, absolute volume difference, CDV, ADV, DSC, and HD), with each observer also compared to the reference delineation. Sample size justification was based on published data reporting a DSC SD of 0.19 in breast CTV_boost_ delineation [5]. With a two-sided alpha of 0.05, a minimal detectable difference of 0.05, and a power >99%, a minimum of 24 patients was required; the inclusion of 58 patients provided 696 intra-patient comparisons, consistent with good-to-excellent reproducibility based on ICC estimates (ICC >0.75–0.90) [14]. To assess systematic differences between strategies, observer-averaged volumes and reproducibility metrics were compared across the four delineation approaches. A subgroup analysis explored whether differences in DSC were influenced by patient or tumor characteristics, including breast size, tumor location, breast density, tumor volume, Ki67 index, and Residual Cancer Burden (RCB). Normality was assessed using the Shapiro–Wilk test; paired comparisons used the Wilcoxon signed-rank test, and subgroup analyses used Student's/Welch's t-tests or Mann–Whitney U tests as appropriate, with p < 0.05 considered statistically significant. Statistical analyses were performed using Python (version 3.10).

## 3 Results

### 3.1 Patients and treatment's characteristics

Patient, tumor, and treatment characteristics are summarized in Table 1. Fifty-eight patients were included in the study, with a mean age of 53.9 years (SD: 12.6; range: 22–74). At initial diagnosis, the majority of tumors were classified as T2 (63.8%), and more than half of the patients (55.2%) presented with lymph node involvement. The predominant histological subtype was invasive ductal carcinoma (89.7%). On the molecular level, 44.2% of tumors were triple-negative, 43.1% were hormone receptor-positive, and 36.2% overexpressed HER2. A high Ki-67 proliferation index was observed in 89.7% of patients.

**Table 1:** Patients, tumor and treatment characteristics (58 patients).

Characteristics	Nb	%
AJCC Stages 8th edition		
cT		
T1b	3	5.2
T1c	13	22.4
T2	37	63.8
T3	5	8.6
cN		
N0	26	44.8
N+	32	55.2
Histological type		
IDC-NST	52	89.6
Medullary carcinoma	1	1.8
Apocrine carcinoma	2	3.4
Poorly differentiated carcinoma	3	5.2
Immunohistochemical Markers		
HR+	25	43.1
HR-	33	56.9
HER2+	21	36.2
HER2-	37	63.8
Ki67 low	1	1.7
Ki67 mid	5	8.6
Ki67 high	52	89.7
Grade		
1	2	3.4
2	14	24.2
3	42	72.4
Breast density		
Fatty (glandular tissue <50%)	41	70.7
Glandular	17	29.3
Tumor site		
Central (central third)	14	24.1
Marginal	44	75.9
Breast size		
Medium	13	22.4
Large (CTV_breast_-TEP >500 mL)	45	77.6
Radiation technique		
3D-CRT	28	48.3
IMRT	30	51.7
Boost Technique		
SIB	26	44.8
Sequential	32	55.2

Abbreviations: IDC-NST: Invasive ductal carcinoma of no special type; HR: Hormone receptor; HER2: Human epidermal growth factor receptor 2; Ki-67: Proliferation index marker; AJCC: American Joint Committee on Cancer; CTV: Clinical target volume; RT: Radiation therapy; 3D-CRT: Three-dimensional conformal radiotherapy; IMRT: Intensity-modulated radiotherapy. SIB: Simultaneous integrated boost.

Tumor laterality was balanced (53.5% left-sided, 46.6% right-sided), with the upper outer quadrant being the most common tumor location (48.3%). Most tumors were histological grade 3 (68.9%), and the majority of patients had fatty or mixed breast density (70.7%). Tumors were considered marginal in 75.9% of cases, and breast volume was medium to large in most patients, with 77.6% presenting a CTV_breast_ (measured on PET-CT) greater than 500 cm^3^.

All patients received PST. A pathological complete response at the primary tumor site (ypT0) was achieved in 48.3% of patients, and 54.4% were classified as RCB0.

The mean time between PET/CT imaging and initiation of PST was 20.3 days (SD: 17.7), and the mean interval between the start of PST (baseline PET/CT) and the start of RT was 7.5 months (SD: 1.2). RT lasted an average of 42.1 days (SD: 9.1). The irradiation technique was three-dimensional conformal radiotherapy in 48.3% of cases and intensity-modulated radiotherapy in 51.7%. A sequential boost was delivered in 55.2% of patients, while a simultaneous integrated boost (SIB) was used in 44.8%.

### 3.2 Contours comparison

The mean volume of the clip-based contours (CTV_CLI_) was 34.4 cm^3^ (95% CI: 29.6–39.2). When comparing this reference anatomy-based strategy to PET-guided approaches, CTV_PET_ showed a similar average volume (36.5 cm^3^; 95% CI: 30.8–42.3; p = 0.93), whereas CTV_INT_ produced larger volumes (43.6 cm^3^; 95% CI: 37.3–49.9; p < 0.001) and CTV_COM_ yielded substantially smaller volumes (21.2 cm^3^; 95% CI: 19.2–23.1; p < 0.001). These volume differences provide the context for the reproducibility analyses summarized in Table 2.

**Table 2:** Interobserver analysis.

Indices Comparison		Mean (95% CI)	Optimal value	*p-value*
**Volume Ratio (VR)**	CTV_CLI_	1.39 (95% CI: 1.16-1.62)	1	ref.
	CTV_PET_	1.14 (95% CI: 1.06-1.21)		
	CTV_COM_	1.02 (95% CI: 1.00-1.03)		
	CTV_INT_	0.88 (95% CI: 0.80-0.96)		
**Common Delineated Volume (CDV)**	CTV_CLI_	0.72 (95% CI: 0.67-0.77)	100%	ref.
	CTV_PET_	0.91(95% CI: 0.87-95)		p < 0.001
	CTV_COM_	0.87 (95% CI: 0.82-0.92)		p < 0.001
	CTV_INT_	0.73 (95% CI: 0.69-0.77)		p = 0.10
**Additional Delineated Volume (ADV)**	CTV_CLI_	0.39 (95% CI: 0.35-0.44)	0%	ref.
	CTV_PET_	0.18 (95% CI: 0.13-0.22)		p < 0.001
	CTV_COM_	0.14 (95% CI: 0.09-0.19)		p < 0.001
	CTV_INT_	0.15 (95% CI: 0.12-0.18)		p < 0.001
**Dice Similarity Coefficient (DSC)**	CTV_CLI_	0.62 (95% CI: 0.58-0.65)	1	ref.
	CTV_PET_	0.86 (95% CI: 0.82-0.90)		p < 0.001
	CTV_COM_	0.86 (95% CI: 0.81-0.91)		p < 0.001
	CTV_INT_	0.77 (95% CI: 0.74-0.80)		p < 0.001
**Hausdorff Distance (HD) (mm)**	CTV_CLI_	16.50 (95% CI: 14.23-18.77)	0	ref.
	CTV_PET_	7.33 (95% CI: 5.33-9.32)		p < 0.001
	CTV_COM_	4.24 (95% CI: 3.00-5.49)		p < 0.001
	CTV_INT_	12.49 (95% CI: 10.60-14.39)		p < 0.001

Compared with the reference contours, the CTV_CLI_ showed the largest deviations across all metrics, with a mean DSC of 0.62 and an HD of 16.5 mm. PET-based approaches showed higher geometric concordance, with DSC values of 0.86 for both CTV_PET_ and CTV_COM_, and lower HD values (7.3 mm and 4.2 mm, respectively). The centroid-based approach (CTV_COM_) showed the smallest discrepancy relative to the reference, with a volume ratio close to 1.0. The integrative approach (CTV_INT_) yielded intermediate values, with a DSC of 0.77 and an HD of 12.5 mm. Detailed quantitative results for all indices are provided in Table 2.

The subgroup analysis did not reveal any significant interaction between patient or tumor characteristics and the reproducibility differences observed between delineation strategies. For all predefined subgroups, the mean DSC differences between CTV_CLI_ and CTV_PET_, CTV_INT_, or CTV_COM_ were not statistically different (all p-values >0.2; see **Sup.** Fig. 1, Fig. 2, Fig. 3).

## 4 Discussion

This study demonstrates that PET/CT-guided delineation strategies can improve inter-observer agreement in defining the CTV_boost_ after PST. Among the four approaches evaluated, PET-informed methods using DIR achieved higher reproducibility than the conventional postoperative approach based on surgical clips and anatomical information. These findings support the use of pretreatment imaging as an additional spatial reference to enhance contouring consistency in this challenging setting.

The choice to focus on patients treated with PST was both pragmatic and clinically motivated. In standard practice, preoperative PET/CT is seldom performed in early-stage or operable breast cancer. Conversely, in patients with locally advanced disease undergoing PST, baseline metabolic imaging is often acquired before any locoregional treatment [10]. This specific population presents a particular challenge for defining the target volume. In cases of major or complete pathological response, postoperative anatomical surrogates, such as the surgical cavity, may no longer accurately represent the spatial extent of the pretreatment tumor. Surgical clips are routinely placed to mark the lumpectomy cavity; however, the challenge in this setting is not related to clip placement itself. Rather, even when correctly positioned, postoperative landmarks may be discordant with the initial tumor location, a situation that has not been specifically quantified in RT studies. This limitation is well recognized in the surgical literature and underlies the use of tumor markers placed before PST to facilitate subsequent localization when the original tumor becomes difficult to identify after response to therapy [7]. Moreover, the indication for boost irradiation in these cases remains unchanged [15], yet the anatomical target is often no longer visible. In this context, the use of pretreatment imaging as an additional spatial reference, rather than as a substitute for postoperative landmarks, may help reduce contouring uncertainty and IOV.

Post-surgical definition of the tumor bed is conceptually and technically challenging, as cavity collapse, fibrosis, and irregular resection fields often lead to poor visibility and inconsistent volume delineation [6]. Moreover, the pathological tumor volume is frequently smaller than both the pre-treatment radiological volume and the surgical specimen, which typically includes wide safety margins. These observations underpin the ongoing debate on whether the CTV_boost_ should reflect the residual tumor bed after PST, as recommended by current guidelines, or whether incorporating pretreatment tumor information could reduce geographic miss at the cost of larger irradiated volumes. In current practice, GEC-ESTRO recommendations remain the reference, defining the CTV_boost_ from the visible lumpectomy cavity or seroma on the planning CT, encompassing surgical clips, with non-isotropic margins derived from the recommended 20-mm margin adjusted to histologically confirmed tumor-free margins [16]**.** However, this approach is complex to implement in practice and is associated with low inter-observer reproducibility, particularly when cavity visibility is poor, as assessed using the Cavity Visualization Score (CVS <3) [4].

In this context, PET-guided strategies reduce exclusive reliance on postoperative anatomical landmarks, particularly when cavity visibility is limited. The centroid-based approach (CTV_COM_) applies guideline-adapted margins around the pre-treatment PET-defined tumor centroid, providing a standardized and clinically realistic estimate of the original tumor location. This method achieved high reproducibility while limiting unnecessary volume expansion. In contrast, CTV_PET_ reproduces the full pre-PST metabolic extent and is therefore less suitable as a clinical boost volume, although it remains informative from a methodological perspective. Nevertheless, PET-guided approaches may be affected by intramammary remodeling after extensive surgery, requiring careful clinical interpretation.

The performance of PET-guided approaches was influenced by technical factors, particularly the use of a 40% SUVmax threshold for BTV segmentation, which, although widely adopted, remains debated [17,18]. This method may be less reliable in low-uptake or heterogeneous tumors and in dense breast tissue, potentially under-representing viable tumor regions. In some cases, manual adjustment of the BTV was required to reconcile metabolic and anatomical discrepancies, highlighting the intrinsic challenges of fusing diagnostic PET/CT with planning CT in the postoperative setting. DIR was used to facilitate anatomical alignment, but its performance was not formally evaluated in this study.

The CTV_INT_ was designed to integrate all available information: clinical context, pathological findings, surgical clip location, and PET metabolic activity. By construction, it seems to provide the most comprehensive and anatomically informed representation of the CTV_boost_. Notably, this resulted in a mean volume (43 mL) slightly larger than the clip-based CTV_CLI_ (34 mL). This observation echoes the broader debate on the optimal balance between completeness of target definition and preservation of normal tissue. In the Danish DBCG Partial Breast Irradiation trial, Thomsen et al. demonstrated a clear dose–volume relationship, with grade 2–3 breast induration occurring from as little as 177 mL of tissue receiving ≥40 Gy [9]. Although CTV_INT_ volumes remained far below this threshold, this finding highlights the need to carefully balance target completeness with normal tissue preservation.

No significant inter-subgroup differences were observed regarding the benefit of PET/CT-guided delineation, with comparable improvements in DSC observed across all predefined categories, including tumor volume, breast density, tumor location, Ki67 index, and RCB. These findings suggest that the added value of PET/CT is not confined to anatomically or biologically distinct contexts. Nevertheless, the consistent gain in contouring reproducibility associated with PET-guided strategies supports their implementation when PET/CT imaging is already performed as part of the PST assessment, particularly to improve boost volume definition in patients undergoing PST.

This study has several strengths, including a blinded multi-observer evaluation and a standardized pipeline for contour comparison and statistical analysis. DIR was systematically applied to facilitate anatomical alignment between PET and planning CT within a consistent workflow. However, several limitations should be acknowledged. The 40% SUVmax threshold may not be optimal across all tumor phenotypes, and manual contour adjustments may introduce observer-dependent variability. The study was not designed to compare PET/CT with preoperative CT alone, which may limit generalizability to centers where baseline PET/CT is not routinely performed. In addition, surgical clips represent the surgical cavity rather than the initial tumor location, PET imaging does not capture intraductal disease, and oncoplastic procedures may alter the spatial relationship between baseline imaging and the definitive tumor bed. These aspects should be considered when interpreting PET-guided approaches.

## 5 Conclusion

This study supports the integration of deformable PET/CT registration into CTV_boost_ delineation workflows for selected breast cancer patients treated with PST. PET-guided approaches demonstrated lower IOV, improving the reliability of boost volume definition, particularly when residual disease is minimal or surgical landmarks are ambiguous. Future work should focus on prospective validation and workflow optimization, including treatment-position PET acquisition. By addressing contouring reproducibility rather than clinical endpoints, these findings provide a methodological framework to better exploit pretreatment imaging when available.

## CRediT authorship contribution statement

**Jordy Kemmeugne:** Writing – original draft, Visualization, Investigation, Data curation. **Lyse Gallay:** Writing – review & editing, Formal analysis. **David Morland:** Writing – review & editing, Investigation. **Judicael Hotton:** Writing – review & editing, Investigation. **Christelle Jouannaud:** Writing – review & editing, Investigation. **Yacine Merrouche:** Writing – review & editing, Investigation. **Dimitri Papathanassiou:** Writing – review & editing, Investigation. **Fabien Reyal:** Writing – review & editing, Investigation. **Marie Ndéo Sene:** Writing – review & editing, Investigation. **Paolo Torielli:** Writing – review & editing, Investigation. **Stéphane Vignot:** Writing – review & editing, Investigation. **Philippe Guilbert:** Writing – review & editing, Validation, Data curation. **Arnaud Beddok:** Writing – review & editing, Visualization, Validation, Supervision, Investigation, Data curation, Conceptualization.

## Data sharing statement

Research data are stored in an institutional repository and will be shared upon request to the corresponding author.

## Declaration of competing interest

The authors have no relevant financial or non-financial interests to disclose.
