# Supplementary material for: Improving CTVboost delineation after preoperative systemic therapy in breast cancer using deformable PET/CT registration
Source: Breast. 2026 Mar 23;87:104752. doi: 10.1016/j.breast.2026.104752 (PMC13068828; doi:10.1016/j.breast.2026.104752)
Supplement: Multimedia component 1 [file mmc1.docx]

Supplementary Material

**PET/CT acquisition and reconstruction details**

Clinical and imaging data were anonymized before analysis. PET/CT scans were acquired 60 minutes after intravenous injection of 3 MBq/kg of FDG, using a Discovery 710 Elite PET/CT system (General Electric, Milwaukee, WI). No contrast agent was used for the CT component (native collimation: 16 x 1.25 mm; auto mA mode with adaptive statistical iterative reconstruction). PET images (2 minutes per bed position) were reconstructed on a 256 × 256 matrix (voxel size: 2.73 × 2.73 × 3.27 mm³) using the VUE Point FX – SharpIR algorithm, which includes corrections for normalization, decay, scatter, attenuation, and integrates time-of-flight and point spread function modeling. An iterative reconstruction algorithm was applied (24 subsets, 2 iterations, Gaussian post-reconstruction filter with a full width at half maximum of 6.4 mm). All PET/CT used in this study corresponded to the pre-treatment baseline imaging performed before PST and therefore represented the metabolic extent of the disease before any therapeutic response.
