# Supplementary material for: Improving CTVboost delineation after preoperative systemic therapy in breast cancer using deformable PET/CT registration
Source: Breast. 2026 Mar 23;87:104752. doi: 10.1016/j.breast.2026.104752 (PMC13068828; doi:10.1016/j.breast.2026.104752)
Supplement: Multimedia component 2 [file mmc2.docx]

**Sup. Figure 1.** **Difference in DSC between CTVCLI and CTVCOM across predefined clinical subgroups.**
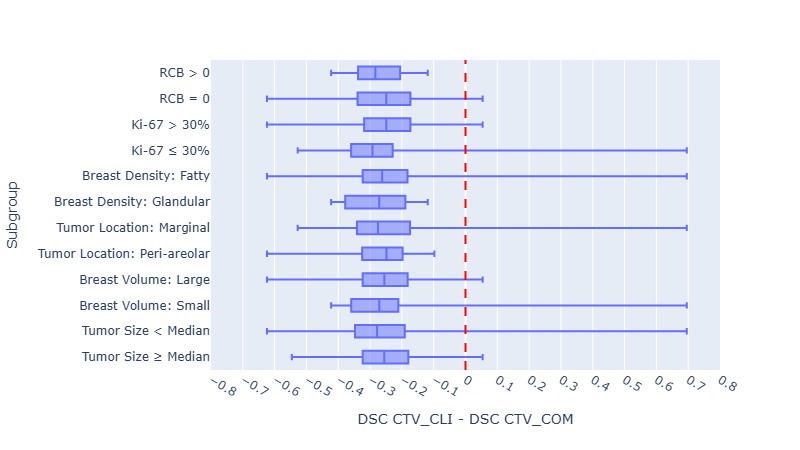


Boxplots represent the distribution of DSC differences (CTVCLI – CTVCOM) for each subgroup. A negative value indicates higher interobserver agreement for CTVCOM than for CTVCLI. Although CTVCOM showed overall better reproducibility than CTVCLI, no subgroup demonstrated a significantly larger or smaller benefit (all p > 0.2).

**Sup. Figure 2.** **Difference in DSC between CTVCLI and CTVINT across predefined clinical subgroups.**

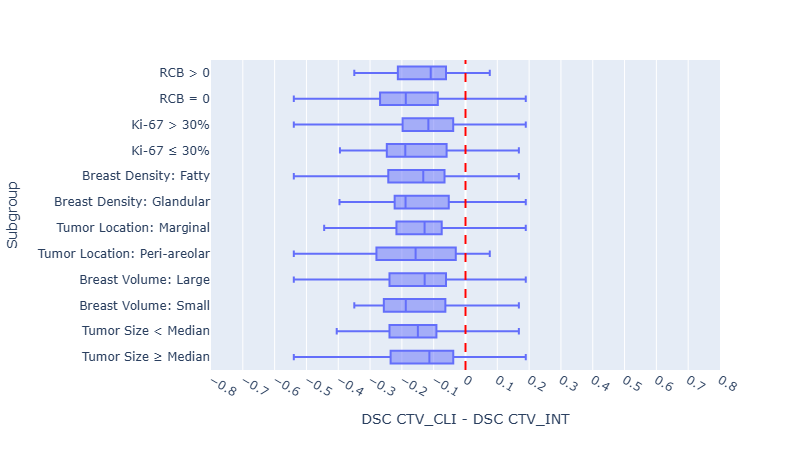


This figure explores whether the reproducibility advantage of CTVINT over CTVCLI varies across patient or tumor characteristics. All subgroup comparisons showed consistent trends, with no statistically significant interaction (all p > 0.2).

**Sup. Figure 3.** **Difference in DSC between CTVCLI and CTVPET across predefined clinical subgroups.**


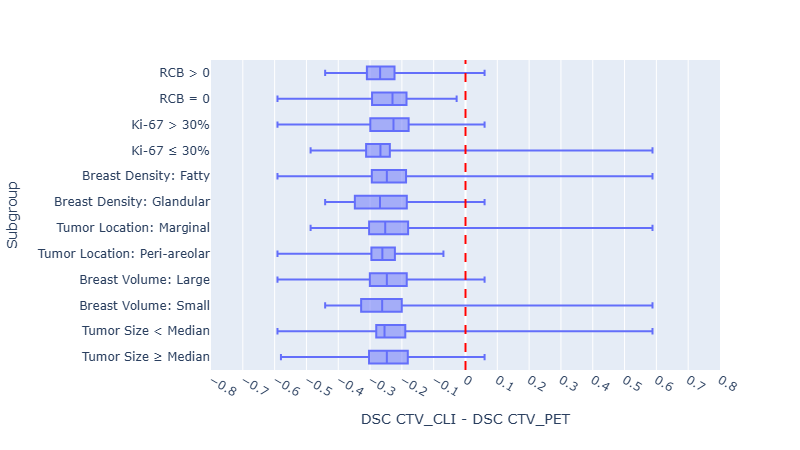
Each boxplot illustrates the DSC difference between CTVCLI and CTVPET within specific clinical or pathological subgroups. Despite overall improved reproducibility with PET-guided delineation, no subgroup exhibited a significantly greater benefit (all p > 0.2).
